# Chemosensory communication of aggression: women's fine-tuned neural processing of male aggression signals

**DOI:** 10.1098/rstb.2019.0270

**Published:** 2020-04-20

**Authors:** Bettina M. Pause, Dunja Storch, Katrin T. Lübke

**Affiliations:** Department of Experimental Psychology, Heinrich-Heine-University Düsseldorf, D-40225 Düsseldorf, Germany

**Keywords:** aggression, body odours, chemosensory communication, olfaction, sex differences

## Abstract

The current study is the first to examine the central nervous processing of aggression chemosignals within men and women by means of chemosensory event-related potential (CSERP) analysis. Axillary sweat was collected from 17 men and 17 women participating in a competitive computer game (aggression condition) and playing a construction game (control condition). Sweat samples were pooled with reference to donor gender and condition, and presented to 23 men and 25 women via a constant flow olfactometer. Ongoing electroencephalogram was recorded from 61 scalp locations, CSERPs (P2, P3-1, P3-2) were analysed and neuronal sources calculated (low-resolution electromagnetic tomography, LORETA). Women, especially, showed larger P3-1 and P3-2 amplitudes in response to male as compared with female aggression signals (all *p* values < 0.01). The peak activation of this effect was related to activity within the dorsomedial prefrontal cortex (Brodmann area 8). As male aggression commonly targets physical harm, the competence of the human brain to sensitively detect male aggression signals is considered to be highly adaptive. The detection of male aggression signals seems to be of higher importance for women than for men. It is suggested that the processing of male aggression signals in women induces an immediate response selection.

This article is part of the Theo Murphy meeting issue ‘Olfactory communication in humans’.

## 1. Introduction

One core function of emotions in social animals is the communication of survival-related behavioural adaptations between conspecifics through social signals [1]. Most widely used signals across the metazoan species are chemosensory in nature [2] and science has just started to uncover their relevance for human behaviour (see [3]). Chemosensory stress signals are ubiquitous in the animal kingdom and seem to act contagiously, alerting group members to potential threats, thereby preventing a direct exposure to the source of danger [4]. In humans, the emotions fear and anxiety can be considered to be part of a stress response [5]. Meanwhile numerous studies demonstrate a successful chemosensory transmission of fear and anxiety in humans (see [6,7]), which, however, sometimes can only be demonstrated in female receivers and is absent in males [8–10].

While intra-species aggression might have evolved in the context of defending or obtaining resources [11], aggressive signals are considered to increase fitness by evoking a defence response in order to avoid an escalated fight [1,12]. In many animal species, scent marks alert conspecifics to the competitive ability or dominance of the signal sender [13,14]. Whether or not the signal perceiver reacts aggressively depends on its own social status and experience, and the context of exposure [15,16]. First studies in humans investigated the communicative properties of chemosignals derived from males' sweat while being engaged in a competitive badminton match [17] or in boxing [18]. Aggression-related chemosignals activate physiological arousal [17], elicit an anxiety-related attentional focus [18] and are processed preferentially within the limbic system [19].

The current study aimed to investigate the central nervous processing of aggression chemosignals. In order to examine early, pre-attentive as well as late, evaluative processes, chemosensory event-related potentials (CSERPs) were recorded [20]. So far, only males' chemosensory aggression signals have been investigated or the related participant samples were too small to investigate gender-related effects in the receivers [17–19]. However, as in humans, the communication of aggression strongly varies with the gender of the signal sender as well as with the gender of the signal perceiver [21,22]; both genders were investigated. It is hypothesized that CSERP responses to human chemosensory aggression signals are indicative of preferential processing.

## 2. Material and methods

### (a) Participants

In total, 50 heterosexual (according to self-labelling) individuals took part in the experiment; however, data of two individuals had to be excluded from analysis owing to pronounced electroencephalogram (EEG) artefacts; see EEG data reduction. The remaining 48 participants (23 males, 25 females) had a mean age of 25.7 years (s.d. = 5.2 years; range = 19–43 years, with age not differing between genders, *p* = 0.266). Participants reported that they were non-smokers, right-handed (Annett Handedness Questionnaire [23]) or both; participants and sweat donors reported that they were of European descent (minimizing effects of culture, ethnos and genetic background). None of the participants reported receiving acute or chronic medication, or the use of drugs. In addition, no participant suffered from any neurological, psychiatric, endocrine or immunological condition, or diseases related to the upper respiratory system. Participating women had a regular menstrual cycle and did not use oral contraceptives. None of these participants acted as a sweat donor in the present experiment.

A brief olfactory screening test revealed no suspicion of general hyposmia in any participant. The test required the participants to detect phenylethyl alcohol (99%, 1 : 100 (v/v) diluted in 1,2-propanediol, 99%; both substances: Sigma-Aldrich, St Louis, Missouri, USA), being present in one of three bottles in two consecutive trials, with the remaining two bottles containing the same volume of solvent (phenylethyl alcohol smells rose-like, and is regularly used as a standard in olfactory sensitivity testing, [24]).

Participants gave their written informed consent and were paid for their participation. The entire study, including the sweat donation procedure, was approved by the ethics committee of the Faculty of Mathematics and Natural Sciences of the Heinrich-Heine-University Düsseldorf (Germany).

### (b) Sweat donation

Methods and results of the sweat donation are presented in detail in the electronic supplementary material (figures S1–S3, tables S1–S3). In brief, axillary sweat was sampled on cotton pads from both armpits of 17 women and 17 men. The donors first attended the aggression induction session, and 1–16 days later, a non-emotional control session. Within the aggression condition, participants were exposed to the Point Subtraction Aggression Paradigm (PSAP, [25,26]). Within this game, the participants' task is to collect as many points as possible via button presses, while a fictitious opponent simultaneously is stealing these points. Participants can choose between three behavioural strategies, one of which is related to overt aggressive behaviour against their opponent. In the control session, the PSAP was replaced by a construction computer game.

Almost all donors (30 out of 34) showed overt aggressive behaviour during the PSAP game. In addition, donors reported a stronger increase of anger during the aggression condition than during the control condition (*p* < 0.001; none of the other basic emotions increased during the aggression condition). Accordingly, their salivary testosterone levels rose during the aggression condition (*p* = 0.05). Donors' mean baseline-corrected heartrate decreased during the control session (*p* = 0.001), but did not change during the aggression condition.

Following the completion of collection, all cotton pads carrying the sweat samples were cut and pooled with respect to the donor's gender and the donation condition. Each of the final four homogenized samples (male aggression, male control, female aggression and female control) was divided into 100 portions of 0.4 g cotton pad and stored at −20°C.

### (c) Presentation of the sweat samples

For EEG recordings and stimulus ratings, the chemosensory stimuli were presented by a constant flow (100 ml s^−1^; stimulus duration = 0.4 s) eight-channel olfactometer (latency of stimulus onset after valve activation = 40 ms; rise time = 50 ms; OL023, Burghart, Wedel, Germany). Both nostrils were stimulated simultaneously, and both air streams were controlled by separate mass flow meters. The temperature of the air flow at the exit of the olfactometer was 37°C and the relative humidity was set above 80%. White noise of 75 dB(A) was presented binaurally via earplugs (Etymotic Research, ER3-14A), in order to prevent the participants from hearing the switching valves of the olfactometer. During EEG recordings and odour ratings, participants performed the velopharyngeal closure technique [27,28].

### (d) Odour detection, odour ratings and emotional ratings

Following each stimulus presentation during the EEG recording, participants indicated whether they had perceived an odour (yes, no), and afterwards (independent of their detection statement), their opinion on whether the putative stimulus was obtained from women or men. Participants indicated either answer by ticking a box on a screen (yes/no or male/female) with a mouse (forced choice). In order not to bias the participants and to ensure attention, participants were told that body odours would only be presented in some, but not all trials. In fact, odours were presented during all trails and no blank trials were included. For odour detection as well as for the assessment of the donors' gender, a hit rate was calculated, defined as percentage of correct answers. Missing data within the detection task were treated as ‘not detected'.

In order to obtain odour ratings, at the beginning of the experiment, before EEG recording, each sample was presented for 0.4 s for each of the three ratings. The order of odour presentations was randomized. Participants rated the sweat samples' intensity on a pictographic scale ranging from 1 (not at all intense) to 9 (extremely intense). In addition, participants selected terms from a list of 147 verbal descriptors that best described the sweat samples' odorous quality [29]. Here, participants were required to select at least one descriptor, but were free to select as many descriptors as they deemed fitting. Participants practised using the descriptor list for as long as they needed to by describing the odour of phenylethyl alcohol, which was used in the hyposmia screening.

In order to assess the donors' emotional experience during donation, participants reported to what extent they thought the donors' felt each of three basic emotions (fear, anger and happiness) on visual analogue scales (0 = ’not at all' to 10 = ’extremely').

### (e) Electroencephalogram procedure

The time course of the entire experimental session, including the EEG procedure, is depicted in electronic supplementary material, figure S4. During EEG recording, each of the four stimuli (male aggression, male control, female aggression and female control) was presented 25 times. The stimuli were presented in a previously randomized, fixed order (with the restriction that the same emotion or the same donor gender was presented no more than three times in a row). Participants were informed that they would receive body odours; however, they knew neither anything about the emotional state of the odour donors, nor how many different odours they would receive. At the beginning of each trial, a fixation cross was presented on a screen for 5.5 s, and sweat samples were presented randomly 2–3 s after cross-onset (stimulus duration: 0.4 s). Subsequent to the fixation cross, the screen turned grey for 2–3 s (randomized), followed by the question ‘Did you smell anything?' appearing on the screen for 3 s. Afterwards, the question ‘Which was the donor's gender?' appeared on the screen for 3 s. In order to ensure sustained attention throughout EEG recording in spite of the relatively long inter-stimulus intervals (ISIs), the participants were further presented with a task during which they had to assign a colour to the odour they just had perceived (3 s). The trials ended with the presentation of a grey screen for 2–5 s (randomized). In total, the trials' duration was 18.5 to 22.5 s (randomized), with a total recording duration of 34 min 10 s. EEG recordings were subdivided into three blocks (33, 33 and 34 trials), separated by two individually adjusted resting periods. On average, the EEG sessions' duration was 41 min (s.d. = 4 min).

### (f) Data recording and reduction

Ongoing EEG was recorded from 61 scalp locations with Ag/AgCl sintered electrodes (inner diameter 6 mm), using an electrode cap (EasyCap, Herrsching, Germany). An additional electrode was placed 1.5 cm below the right eye, outside the vertical pupil axis to record the vertical eye movements. Fp2 was used to record the horizontal eye movements. The ground electrode was placed at position FT10. The electrodes' impedance was usually below 10 and always below 20 kΩ. Data were sampled at 500 Hz with an averaged reference and low-pass filtered online at 135 Hz (QuickAmp-72 amplifier and BrainVision Recorder software, Brain Products, Munich, Germany).

Offline, EEG signals were re-referenced to linked ear lobes, low-pass filtered with 40 Hz (48 dB/octave) and high-pass filtered with 0.05 Hz (48 dB/octave). Additionally, a 50 Hz notch filter was applied. Each EEG was corrected for eye movements [30] and baseline-corrected (500–0 ms before stimulus onset). Channels containing voltage bursts (75 µV maximum voltage difference within 100 ms) were excluded from the analyses. In cases where more than one-third of the channels forming one electrode pool (see below) were contaminated with artefacts in a given trial, trials were also excluded. In sum, two participants were completely excluded from analysis (with fewer than 13 out of 25 trials in at least one condition).

For peak detection, the artefact-reduced EEG was low-pass filtered with 7 Hz, 48 dB/octave. The 61 scalp electrode positions were subdivided into nine areas (pools), and a mean peak for each pool was calculated by averaging adjacent electrodes in anterior (a), central (c) and posterior (p) areas for the left (l) and the right (r) hemisphere as well as for midline electrodes (resulting electrode pools: al: AF7, AF3, F7, F5, F3; am: Fpz, AFz,F1, Fz, F2; ar: AF4, AF8, F4, F6, F8; cl: FT7, FC5, FC3, T7, C5, C3, TP7, CP5, CP3; cm: FC1, FCz, FC2, C1, Cz, C2, CP1, CPz, CP2; cr: FC4, FC6, FT8, C4, C6, T8, CP4, CP6, TP8; pl: P7, P5, P3, PO7, PO3, O1; pm: P1, Pz, P2, POz, Oz; pr: P4, P6, P8, PO4, PO8, O2). In relation to the baseline period (500–0 ms before stimulus onset), four separate peaks were differentiated within predefined latency windows (N1: 250–600 ms, P2: 500–700 ms, P3-1: 700–900 ms, P3-2: 900-1100 ms; [20]), and amplitudes and latencies of each peak were calculated. As the N1 deflection within the present data was almost absent (mean, *M* = −0.4 µV, s.d. = 1.1), we refrained from statistically analysing the N1.

### (g) Data analysis

Detection rates, odour intensity and the attribution of the donors' gender were analysed by means of three-way mixed-factors ANOVAs, including the within-subjects factors Emotion (EMO; aggression sweat sample, control sweat sample), Donors' Gender (DG; male sweat sample, female sweat sample) and the between-subjects factor Participants' Gender: (PG; men, women). Detection rates for each sweat sample (male aggression, male control, female aggression and female control) were also tested against chance level by means of one-sample *t*-tests. In order to investigate whether participants could identify the emotional content of the sweat samples, the suspected emotions of the donors were analysed by means of a two-way mixed-factors ANOVA separately for each sweat sample, including the within-subjects factor Assessed Emotion (anger, fear and happiness) and the between-subjects factor PG. All significant ANOVA results regarding the detection rates and ratings are reported.

The amplitudes and latencies of the CSERP components were subjected to a five-way mixed-factors ANOVA, including the within-subjects factors EMO, DG, Sagittal (SAG; anterior, central, posterior) and Transversal (TRANS; left, midline, right), and the between-subjects factor PG. Significant interactions were followed up by nested ANOVA effects analysis [31] and, in the case of significant nested effects, simple comparisons (e.g. paired *t*-tests). In all analyses, the alpha level was set to *p* < 0.05 (based on Huynh–Feldt corrected degrees of freedom). Within the main article, all significant ANOVA and nested ANOVA effects including the factors EMO, DG and PG are reported. Effects including exclusively the factors SAG and TRANS are reported in the electronic supplementary material.

Current source density (CSD) maps were calculated using a spherical spline model ([32], order of splines: *m* = 4, maximal degree of Legendre polynominals = 20). Low-resolution electromagnetic tomography (LORETA) was used in order to localize the source of brain activity [33]. The source space comprises 2394 voxels at 7 mm spatial resolution, covering the cortical grey matter and the hippocampus [34], defined via a reference brain from the Brain Imaging Center at the Montreal Neurological Institute (MNI, [35]). LORETA uses a three-shell spherical head model, co-registered to the Talairach anatomical brain atlas [36].

## 3. Results

### (a) Stimulus detection and assessment of donors' gender

During EEG recording, participants detected on average 52.3% (s.d. = 26.7; range = 0.0–100.0%) of the presented sweat samples, not differing from chance in their overall detection performance (*t*_47_ = 0.60, *p* = 0.555). However, separating detection rates for each stimulus, male aggression sweat was detected more often than expected by chance (*M* = 60.0%, s.d. = 30.0; *t*_47_ = 2.31, *p* = 0.025). Odour detection rates did not change from the first to the second 50 trials (*t*_47_ = 0.90, *p* = 0.375).

In general, detection rates were higher for male (*M* = 56.0%, s.d. = 27.2) than for female sweat (*M* = 48.5%, s.d. = 27.4; DG: *F*_1,46_ = 20.16, *p* < 0.001, $\eta_{p}^{2}=0.31$, power = 0.99), and women responded more often to aggression sweat (*M* = 56.2%, s.d. = 28.2) than to control sweat (*M* = 46.4%, s.d. = 27.5; EMO × PG: *F*_1,46_ = 6.86, *p* = 0.012, $\eta_{p}^{2}=0.13$ , power = 0.73; nested effects: EMO within women: *F*_1,46_ = 15.68, *p* < 0.001).

Participants' correct assessment of the donors' gender did not differ from chance (*p* = 0.066). On average, participants correctly assessed 51.6% (s.d. = 5.9) of the presented samples. Neither participants' gender nor the chemosensory condition affected the assessment (all *p* values > 0.089). All group mean values regarding stimulus detection (table S4) and donors' gender assessment (table S5) are presented in the electronic supplementary material.

### (b) Odour ratings and descriptions

#### (i) Intensity

Across all samples, the body odours' intensity was judged as relatively weak (*M* = 3.02, s.d. = 1.54), with male sweat (*M* = 3.33, s.d. = 1.76) being judged as slightly more intense than female sweat (*M* = 2.70, s.d. = 1.59; DG: *F*_1,46_ = 10.33, *p* = 0.002, $\eta_{p}^{2}=0.18$, power = 0.88). However, intensity ratings were unaffected by the emotional condition or participants' gender (all *p* values > 0.142; for all group mean values see electronic supplementary material, table S6).

#### (ii) Suspicion of donors' emotional state

In general, any emotion the participants suspected the sweat donors to have experienced during sweat donations was rated as very low in intensity (*M* = 1.88, s.d. = 1.39). Participants imagined the donors of male aggression sweat to have been more anxious (*M* = 2.57, s.d. = 2.64) than happy (*M* = 1.28, s.d. = 1.61; Assessed Emotion: *F*_2, 88_ = 5.34, *p* = 0007, $\eta_{p}^{2}=0.11$, power = 0.82). Ratings did not differ in the context of any other sweat sample and were not affected by the raters' gender (all *p* values > 0.050; for all group mean values see electronic supplementary material, table S7).

#### (iii) Verbal descriptors

Out of the 147 verbal descriptors the participants could choose from, they selected the descriptor ‘light' most often, and the descriptor ‘warm' second most often for characterizing each of the four sweat samples (for the frequency distribution of selected verbal descriptors see electronic supplementary material, figures S5 and S6).

### (c) Chemosensory event-related potentials

**Figure 1.**
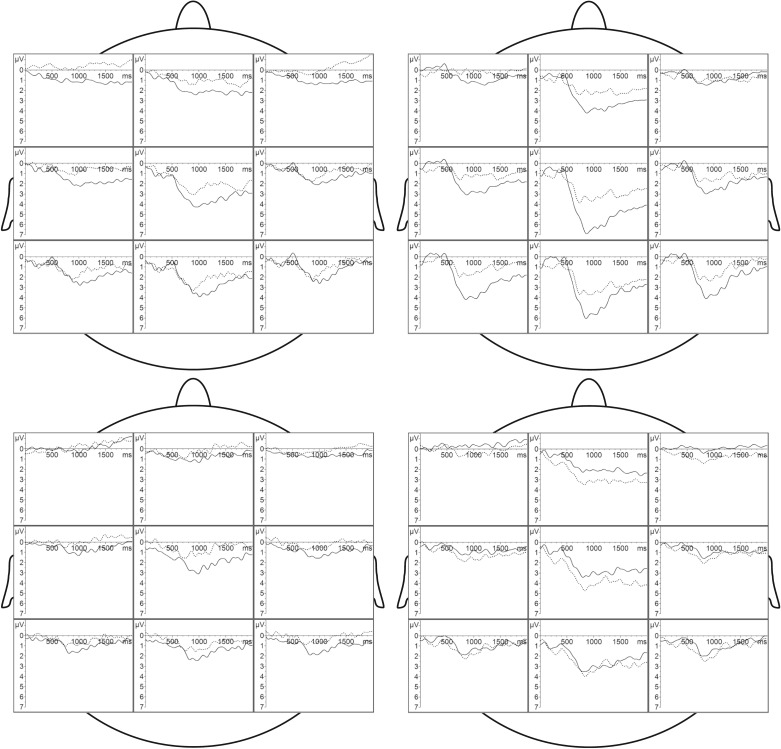
Grand averages of the CSERP across male (left column) and female (right column) participants in response to male (upper row) and female (lower row) sweat. Black lines indicate CSERPs for aggression sweat and dotted lines indicate CSERPs for control sweat. Time point 0 refers to the valve activation.

The distribution of CERPs across the scalp, separated for the experimental conditions, is depicted in figure 1. All CSERP ANOVA effects are listed in electronic supplementary material, tables S8 and S9. A detailed analysis of the CSERP components' local distribution is included in the electronic supplementary material.

#### (i) Amplitudes

*P2-amplitude.* When presented with aggression sweat, participants display larger P2 amplitudes in response to male (*M* = 2.30 µV, s.d. = 2.39) as compared with female sweat samples (*M* = 1.49 µV, s.d. = 1.88; EMO × DG: *F*_1,46_ = 4.41, *p* = 0.041, $\eta_{p}^{2}=0.09$, power = 0.54; nested effects: DG within aggression sweat: *F*_1,46_ = 4.81, *p* = 0.033, $\eta_{p}^{2}=0.09$, power = 0.57).

*P3-1 amplitude.* The amplitude of the P3-1 component is affected by the donors' emotion, the donors' gender and the participants' gender: female participants' P3-1 amplitude is larger in response to male aggression sweat than to male control sweat (EMO × DG × PG: *F*_1,46_ = 6.14, *p* = 0.017, $\eta_{p}^{2}=0.12$, power = 0.68; nested effects: EMO within male sweat within women: *F*_1,46_ = 9.82, *p* = 0.003, $\eta_{p}^{2}=0.18$, power = 0.87; male aggression sweat: *M* = 4.26 µV, s.d. = 3.40; male control sweat: *M* = 2.56 µV, s.d. = 2.06; restricting the first-order interaction EMO × DG to female participants, and reducing the relevance of the EMO × DG × TRANS interaction).

Furthermore, female participants show a larger P3-1 amplitude in response to male aggression sweat than to female aggression sweat (based on the same interaction EMO × DG × PG; nested effects: DG within female participants within aggression sweat: *F*_1,46_ = 12.15, *p* = 0.001; $\eta_{p}^{2}=0.21$, power = 0.93; male aggression sweat: *M* = 4.26 µV, s.d. = 3.40, female aggression sweat: *M* = 2.41 µV, s.d. = 2.50; accordingly, the main effect DG and the interaction EMO × DG are limited to the significant second-order interaction).

Finally, female participants display larger P3-1 amplitudes than male participants in response to female control sweat (based on the same interaction EMO × DG × PG; nested effects: PG within female sweat within neutral sweat: *F*_1,46_ = 5.32, *p* = 0.026, $\eta_{p}^{2}=0.10$, power = 0.61; women: *M* = 3.13 µV, s.d. = 2.26, men: *M* = 1.55 µV, s.d. = 2.49; invalidating the first-order interaction PG × TRANS).

*P3-2 amplitude.* Within the P3-2 latency range, female participants respond with a larger amplitude to male aggression as compared with male control sweat (EMO × DG × PG: *F*_1,46_ = 4.61, *p* = 0.037, $\eta_{p}^{2}=0.09$, power = 0.55; nested effects: EMO within male sweat within women: *F*_1,46_ = 7.21, *p* = 0.010, $\eta_{p}^{2}=0.14$, power = 0.75; male aggression sweat: *M* = 4.08 µV, s.d. = 3.34, male control sweat: *M* = 2.61 µV, s.d. = 2.30). Men, on the other hand, show a significant emotion-specific P3-2 amplitude only in response to female sweat (EMO × DG × PG; nested effects: EMO within female sweat within men: *F*_1,46_ = 4.50, *p* = 0.039, $\eta_{p}^{2}=0.09$, power = 0.55; female aggression sweat: *M* = 2.05 µV, s.d. = 1.62, female control sweat: *M* = 1.10 µV, s.d. = 2.46; invalidating a general implication of the main effect EMO and the first-order interaction EMO × DG).

Moreover, in female participants, P3-2 amplitudes in response to male aggression sweat are larger as compared with P3-2 amplitudes in response to female aggression sweat (EMO × DG × PG; nested effects: DG within women within aggression sweat: *F*_1,46_ = 15.07, *p* < 0.001, $\eta_{p}^{2}=0.25$, power = 0.97; male aggression sweat: *M* = 4.08, µV, s.d. = 3.34, female aggression sweat: *M* = 1.93 µV, s.d. = 2.49; accordingly, the main effect DG and the interaction EMO × DG are limited to the significant second-order interaction).

Indeed, similar to the P3-1, women generally display larger P3-2 amplitudes than men in response to female control sweat (EMO × DG × PG; nested effects: PG within female sweat within neutral sweat: *F*_1,46_ = 5.09, *p* = 0.029, $\eta_{p}^{2}=0.10$, power = 0.60; women: *M* = 2.72 µV, s.d. = 2.51, men: *M* = 1.10 µV, s.d. = 2.46; invalidating the first-order interaction PG × TRANS).

#### (ii) Latencies

The P2 latency is not affected by any experimental condition (all *p* values > 0.057). However, both P3-1 and P3-2 latencies vary with the sweat samples' emotional content as well as the donors' gender.

The P3-1 latency is larger in response to male aggression as compared with male control sweat at left electrode positions (EMO × DG × TRANS: *F*_2,92_ = 8.43, *p* = 0.002, $\eta_{p}^{2}=0.16$, power = 0.96; nested effects: EMO within left pools within male sweat: *F*_1,46_ = 9.32, *p* = 0.004, $\eta_{p}^{2}=0.17$, power = 0.85; aggression sweat: *M* = 825.76 ms, s.d. = 50.81, control sweat: *M* = 796.44 ms, s.d. = 47.82). The P3-2 shows a similar pattern, generally appearing with a longer latency upon presentation of male aggression sweat (*M* = 1015.48 ms, s.d. = 46.77) as compared with male control sweat (*M* = 989.47 ms, s.d. = 46.94; EMO × DG: *F*_1,46_ = 15.70, *p* < 0.001, $\eta_{p}^{2}=00.25$, power = 0.97; nested effects: EMO within male sweat: *F*_1,46_ = 10.48, *p* = 0.002; $\eta_{p}^{2}=0.19$, power = 0.89).

In response to female sweat, however, the P3-2 latency shows the reverse pattern, with a longer latency in response to female control (*M* = 1012.59 ms, s.d. = 48.46) as compared with female aggression sweat (*M* = 988.22 ms, s.d. = 49.34; EMO × DG: *F*_1,46_ = 15.70, *p* < 0.001, $\eta_{p}^{2}=0.254$, power = 0.972; nested effects: EMO within female sweat: *F*_1,46_ = 5.27, *p* = 0.026, $\eta_{p}^{2}=0\;.10$, power = 0.61).

Finally, after presentation of aggression sweat, the P3-2 latency in response to male sweat (*M* = 1015.48 ms, s.d. = 46.77) is larger than in response to female sweat (*M* = 988.22 ms, s.d. = 49.34), but the reverse is true in the case of control sweat (male control sweat: *M* = 989.47 ms, s.d. = 46.94; female control sweat: *M* = 1012.59 ms, s.d. = 48.46; EMO × DG: *F*_1,46_ = 15.70, *p* < 0.001, $\eta_{p}^{2}=0.25$, power = 0.97; nested effects: DG within aggression sweat: *F*_1,46_ = 6.79, *p* = 0.012, $\eta_{p}^{2}=0.13$, power = 0.72; DG within control sweat: *F*_1,46_ = 6.50, *p* = 0.014, $\eta_{p}^{2}=0.12$, power = 0.70).

### (d) Current source density analyses

**Figure 2.**
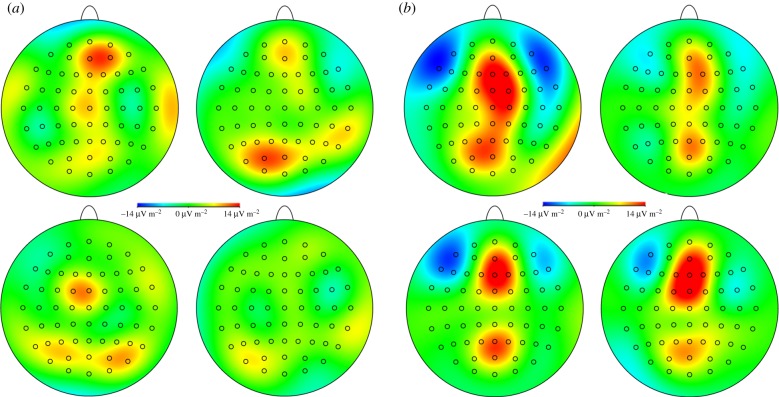
CSD maps (two-dimensional smoothing for a view across all electrodes) at the time of the total mean P3-1 peak latency (810 ms). (*a*) CSD maps of men in response to male aggression sweat (upper left), male control sweat (upper right), female aggression sweat (lower left) and female control sweat (lower right). (*b*) CSD maps of women in response to male aggression sweat (upper left), male control sweat (upper right), female aggression sweat (lower left) and female control sweat (lower right). Red colours represent cortical activation (neuronal sources) and blue colours represent cortical deactivation (neuronal sinks).

Within the P3-1 latency range, men respond to male aggression sweat with cortical activations along the midline, strongest at frontopolar brain areas (figure 2*a*). In response to male control sweat, a left-sided parieto-occipital activation is dominant. Men's brain responses to female sweat in general are weaker than to male sweat. In response to female aggression sweat, parietal areas are bilaterally activated. Neuronal responses to female control sweat appear extremely weak and disperse.

Women show a pattern of cortical activation along the midline, with distinct clusters of activation across frontocentral and parietal areas in response to all sweat samples (figure 2*b*). Simultaneously, inhibition is prominent bilaterally across fronto-temporal areas. This pattern of activation is most pronounced in response to male aggression sweat.

**Figure 3.**
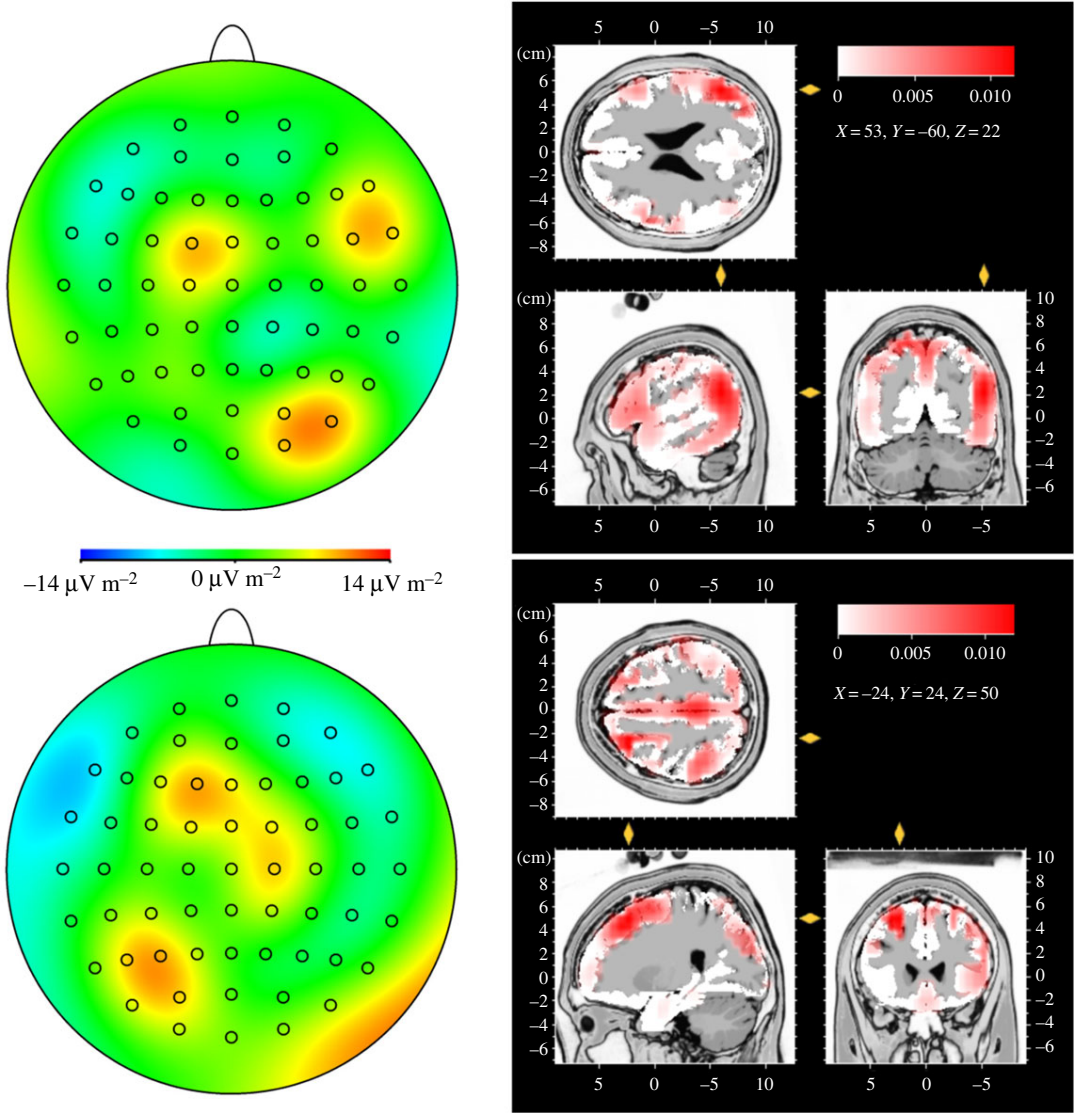
CSD difference maps (two-dimensional smoothing for a view across all electrodes) of differential CSERPs of male participants in response to female aggression minus female control sweat (left, top), and female participants in response to male aggression minus male control sweat (left, bottom) at the time of the total mean P3-1 peak latency (810 ms). Red colours represent cortical activation (neuronal sources) and blue colours represent cortical deactivation (neuronal sinks). LORETA maps depicting the location of the maximum current density (in µA mm^−2^) at the time of the total mean P3-1 peak latency (810 ms) of men responding to female aggression sweat (in contrast with female control sweat, right, top), and women responding to male aggression sweat (in contrast with male control sweat, right, bottom).

With regard to the CSERP results, an emotion-specific differential brain response could be observed when males were smelling female sweat and when females were smelling male sweat. Accordingly, CSD difference maps (aggression–control) were calculated for the respective conditions (figure 3). In males smelling females, aggression-specific activity seems to be most prominent in right parietal brain areas. In females smelling males, aggression-specific activity appears to be most prominent above left frontal brain areas (CSD difference maps for all experimental conditions are plotted in electronic supplementary material, figure S7).

### (e) Low-resolution electromagnetic tomography

LORETA analyses are limited to the conditions resembling significant emotion-related effects (males smelling female sweat and females smelling male sweat, figure 3). In males, the peak activation in response to female aggression sweat (as difference in relation to female control sweat) appears within the right angular gyrus (Brodmann area, BA 39). In females, the maximum activation in response to male aggression sweat (as difference in relation to male control sweat) can be observed in the dorsomedial frontal gyrus (BA 8, LORETA analyses for all difference (aggression–control) conditions are shown in the electronic supplementary material, figure S8).

## 4. Discussion

The current study is the first to our knowledge to show enhanced neural processing of human aggression sweat. It is found that male aggression signals are more intensely processed than female aggression signals and that especially women's brains respond strongly to male aggression signals. These effects seem unlikely to be consciously mediated, as the sweat samples could hardly be recognized as odours.

The sweat was obtained from odour donors experiencing a strong increase in anger during being frustrated by a fictitious co-player. The increase of anger is a valid indicator of reactive aggression [37] and occurred emotion-specifically (no other emotion increased simultaneously). The anger increase was accompanied by an increase of testosterone, as typically associated with PSAP-induced aggressive behaviour [26]. Accordingly, almost all sweat donors reacted with overt aggressive behaviour towards their opponent.

Sweat samples from the aggression condition were rated as equally low in intensity to the control sweat samples, and both were described as predominantly light and warm. The use of the descriptor ‘warm' might refer to the air flow being presented by the olfactometer at body temperature; the predominant use of ‘light' seems to reflect a non-specific and faint odour perception. Further, participants were not able to assign the correct gender or emotion to the donors of the sweat samples. However, across all participants, male aggression sweat was the only stimulus detected as an odour, while detection rates of all other stimuli did not differ from chance. Since the participants were aware of their constant connection to an olfactometer, they might have expected to receive olfactory stimuli at least in certain trails. Thus, we consider the participants to probably have been biased towards reporting smelling an odour, rather than reporting no odour perception. Accordingly, the detection rates reported can be considered to overestimate the true detection performance. It is concluded that the stimuli were perceived at the level of the perceptual threshold, not being associated with a specific odour quality profile. Even though being processed as relevant information in the human brain, human emotional chemosensory stimuli have repeatedly been reported to be difficult to detect or to recognize (e.g. [9,19]). However, in the present study, male aggression sweat was the only stimulus recognized as odorous more frequently than would be expected by chance.

In accordance with the higher detection rates for male aggression odour, the respective chemosensory signal evoked a larger P2 amplitude and longer P3-1 and P3-2 latencies than all other stimuli. As prior work on the chemosensory communication of dominance or aggression relied on male chemosignals only [17–19], this is the first study to our knowledge demonstrating the strong impact of male aggression signals on the human brain. The chemosensory P2 amplitude is an indicator of pre-attentive processes and is affected by the stimulus intensity [38]; therefore, its increased amplitude in response to male aggression signals might reflect the stronger odour of male aggression signals and their capacity to catch neuronal resources. The P3, on the other hand, reflects late evaluative stimulus processing and is not related to exogenous stimulus properties, but to the subjective stimulus relevance [20]. As aggression signals do not automatically induce a certain response, but might evoke fight or flight responses depending on the perceivers' own competencies, response selection strategies need to be carefully balanced [22]. Accordingly, a prolonged P3 latency has been described to be due to effortful response selection strategies [39]. Male aggression is most often expressed as physical aggression [40] and thus can threaten physical health or can even be life threatening. Successful survival depends on a sensitive detection of such signals.

In addition to the general effects of male aggression chemosignals on the P2-amplitude and P3-1 and P3-2 latencies in male and female perceivers, the most pronounced effects on the P3-1 and P3-2 amplitudes can be observed in female participants. Within the P3 latency range, women show larger potentials (P3-1, P3-2) than men. They especially respond to male aggression sweat with much larger potentials (P3-1, P3-2) than to male control sweat or to female aggression sweat. These findings are in line with a female processing advantage of chemosensory anxiety signals [8–10], and suggest a general superior processing of human emotion-related chemosignals in women. The CSERP effects are accompanied by neuronal sources within medio-frontocentral brain regions and neuronal inhibition within fronto-lateral regions (CSD maps). However, similar but weaker activations appear in women in response to all sweat samples. It is suggested that these findings reflect the activation of the mirror neuron system, indicative of contagious effects of social emotions [41], and the inhibition of brain structures related to higher-order reasoning, such as executive functions (dorsolateral prefrontal cortex, [42]). Brain activity specifically related to male aggression signals in women is supposedly located in the dorsomedial prefrontal cortex (DMPFC, BA 8, LORETA difference maps). Activation of the DMPFC seems to be intimately connected to social cognition and is considered to be involved in a self-referential evaluation of others [43], and in the translation of negative social experiences into threat-related physiological responding [44]. Thus, as indicated by the prolonged P3 latencies and the LORETA analyses, male aggression sweat warrants not only a fine-tuned sensory analysis, but in addition an immediate response selection. This is especially important for women, as globally, about one-third of ever-partnered women have experienced physical and/or sexual intimate partner violence [45].

Men, however, respond to a lesser extent to all sweat samples, but still do show a differential brain response to aggression as contrasted to control sweat. This response occurs at a relatively late processing stage (P3-2) and is more prominent in response to female sweat. However, as male participants show almost no response to female control sweat, the significant difference is due to the fact that they still show a slight response to female aggression signals. A heightened sensitivity to same-sex aggression in males, as proposed by some authors [22], could not be statistically confirmed by the present data. However, a weak differentiation of male aggression signals from male control signals in male participants is suggested by visual inspection of the grand averages (figure 1) and direct effect testing (P3-2 amplitude: EMO within male sweat within men *F*_1,46_ = 4.14, *p* = 0.048). Whereas brain responses to male aggression sweat in females could be partly due to the fact that male aggression sweat was slightly odorous but male control sweat was not, the brain responses to female aggression sweat in male participants cannot be explained by any odour-related effects.

In conclusion, chemosensory aggression signals, derived from highly angry and aggressively behaving sweat donors, were obtained from and presented to both genders. The sweat samples were only weakly odorous, they failed to convey a distinct odour quality profile, and intensity ratings were not associated with the emotional state of the odour donors. The human brain strongly responds to male aggression signals, and, especially in women, a pattern of distinct activated and deactivated neuronal assemblies can be observed. Thus, in contrast to chemosensory anxiety signals, the meaning of chemosensory aggression signals varies with the gender of signal sender and perceiver. It is hypothesized that aggression signals not only need to be processed preferentially, but also prompt immediate response selection strategies, in order for the perceiver to be able to cope with the potential threat. As chemosensory communication in humans is far less prone to the effects of learning and culture than any other kind of communication, it is further suggested that the investigation of human chemosensory communication offers a unique but easy way to understand social behaviour in biologically relevant settings.

## Supplementary Material

Sweat donation and main experiment

## Supplementary Material

Original data: Sweat donation

## Supplementary Material

Original data: Main experiment

## Data accessibility

All original data reported in the study can be accessed via the electronic supplementary material.
