# Supplementary material for: Chemosensory communication of aggression: women's fine-tuned neural processing of male aggression signals
Source: Philos Trans R Soc Lond B Biol Sci. 2020 Apr 20;375(1800):20190270. doi: 10.1098/rstb.2019.0270 (PMC7209929; doi:10.1098/rstb.2019.0270)
Supplement: Sweat donation and main experiment [file rstb20190270supp1.pdf]

Superior cortical processing of men's aggression chemosignals in women

Supplementary Material

PART A: SWEAT DONATION

PART B: MAIN EXPERIMENT

Bettina M. Pause, Dunja Storch, Katrin T. Lübke

Department of Experimental Psychology, Heinrich-Heine-University Düsseldorf, D-40225 Düsseldorf, Germany

Correspondence:

Bettina M. Pause

Department of Experimental Psychology, Heinrich-Heine-University Duesseldorf

Universitaetsstrasse 1, D-40225 Duesseldorf, FRG

Tel.: +49 211-81-14384, Fax: +49 211-80-12019

Email: bettina.pause@hhu.de

PART A: SWEAT DONATION

1. Methods

Seventeen women and 17 men (mean age = 23.7 years, SD = 4.9, range = 18-44, with no significant difference between men and women, p = .389) were recruited as sweat donors. Their body-mass-index ranged from 18.0 to 28.6 kg/m^2 (M = 22.6, SD = 2.6). All sweat donors reported being non-smokers and of European origin, and denied any acute or chronic medication. No donor indicated suffering from any neurological, psychiatric, endocrine, or immunological disease, or using drugs. All donors shaved their axillary hair 2 days before each session. They were advised to wash their armpits exclusively with an odourless medical soap (Eubos®, Dr. Hobein GmbH, Germany) the day before, and only with water the day of the body odour donation. Further, they were instructed to refrain from eating garlic, onions, asparagus, or any other spicy or aromatic food during 24 h prior to the sweat donation. Female donors either reported having a regular menstrual cycle (n = 5) or using oral contraceptives (n = 12). Naturally cycling women were required to be in the follicular phase of their menstrual cycle in both donation conditions. All donors gave written informed consent, and were paid for their donation.

Axillary sweat was sampled on cotton pads (ebelin cotton pads, dm-drogerie markt GmbH & CoKG, Karlsruhe, Germany) from both armpits for a duration of 94.4 min (SD = 8.3). The donors first attended the aggression induction session, and 1-16 (M = 4.4, SD = 3.8) days later a non-emotional control session. As the order of sessions was not counterbalanced, all odour donors were treated equal. Emotional carry over effects were precluded, because the sessions were separated by at least one day. Within the aggression condition, participants' thoughts were primed for aggressive contents by presenting video clips of aggressive encounters (duration: 17 min; e.g. boxing, street fights, martial art fights). Afterwards, the participants were exposed to the Point Subtraction Aggression Paradigm (PSAP, [1, 2]). The

PSAP is a computer based game (programmed using Presentation 16.7, Neurobehavioral Systems, USA), divided into three blocks à 12 minutes separated by two breaks of two minutes each (figure S1). Within this game, the participants' task is to collect as many points as possible via button presses, while a fictitious opponent simultaneously is stealing these points (frustration, one point was subtracted from the donors' account randomly every 6 to 60 seconds). Participants can choose between three behavioural strategies: By pressing button A 100 times, one point is added to their account (gain orientated behaviour). By pressing button B 10 times, one point is deleted from their opponents account (aggressive behaviour), and 10 presses of button C saves the participants account for a short period of time (withdrawal-related behaviour; frustration free interval varied randomly between 60 and 120 seconds). At the end of the session, PSAP points could be exchanged to euros (1 point = 0.20 €). None of the donors stated any concern regarding the cover story (inquired by open questions on the interaction with the fictional co-player; e.g. "What is your opinion about your co-player?").

In the control session, the priming video clips were replaced by clips extracted from a documentary science movie on the origin of life (17 min), and the PSAP was replaced by a construction computer game (Lego Digital Designer 4.3, LEGO Group, Denmark, 40 min). Participants were instructed to play the construction game in order to evaluate the game. Figure S1 represents the structure of the aggression and the control condition, including psychological and physiological measurements.

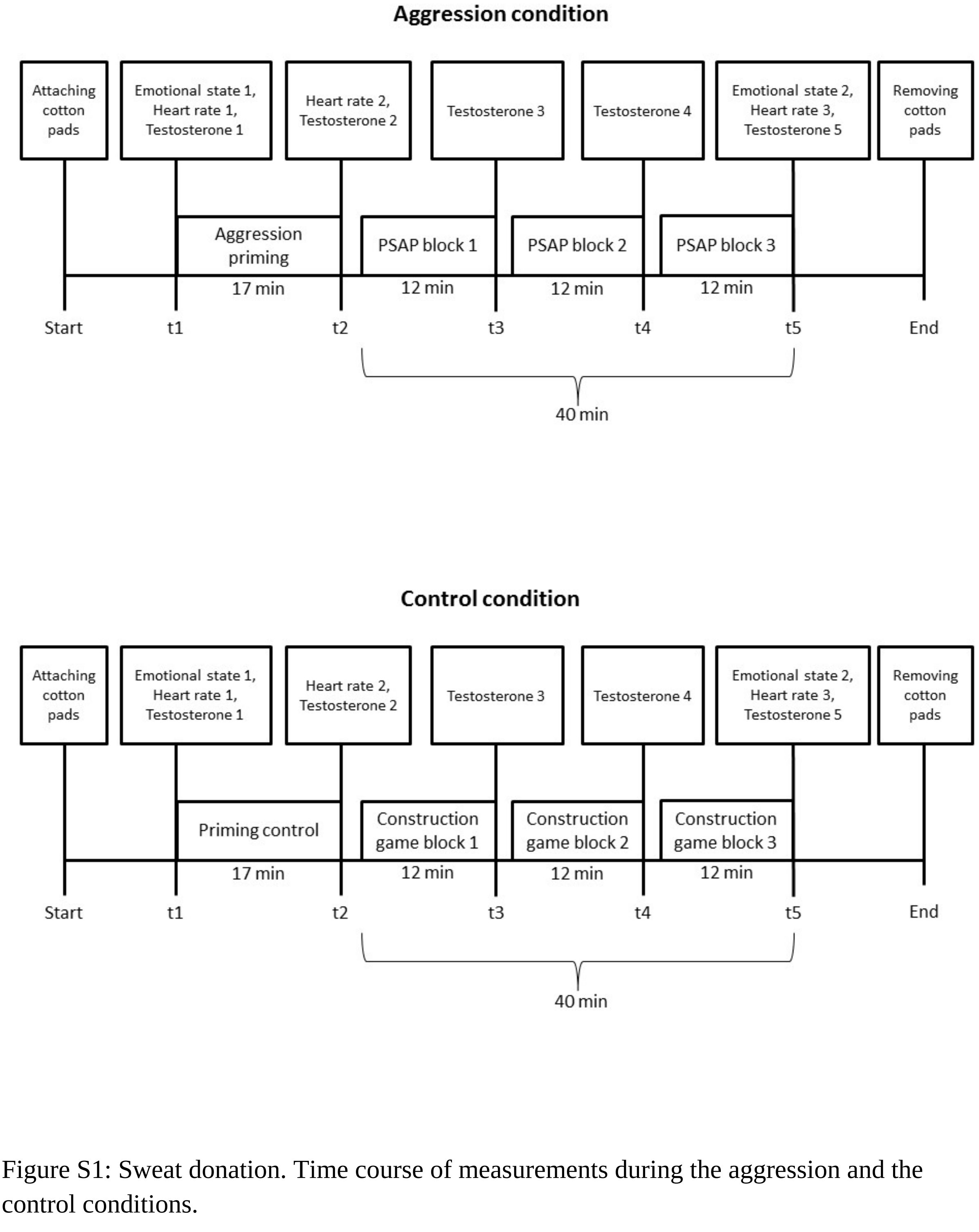

Figure S1: Sweat donation. Time course of measurements during the aggression and the control conditions.

2. Results

Almost all donors (30 out of 34; 15 male, 15 female) showed overt aggressive behaviour (M = 17.2 % of the intra-individual behaviour, SD = 13.8%) by hitting button B during the PSAP game.

Donors reported their emotional state via six visual analogue scales (anger, disgust, fear, happiness, sadness, surprise; 0 = “not at all” to 10 = “extremely”) at the beginning of each session and after the PSAP or the construction game, respectively (figure S1). Donors reported a stronger increase of anger during the aggression condition (M = +1.96, SD =1.88) than during the control condition (M = -0.16, SD = 1.34; SESSION: $F(1, 32) = 37.96$, $p < .001$, $\eta^2_p = .54$, Power: 1.00; see figure S2). During the aggression condition, self-reported anxiety decreased to a stronger degree (M = -0.66, SD = 1.17) than during the control condition (M = -0.34, SD =0.79; SESSION: $F(1, 32) = 4.30$, $p = .046$, $\eta^2_p = .12$, Power: .52). All other emotions were not differently affected by either condition (see table S1 for the mean values of each emotion, table S2 for all ANOVA results, and figure S2 for a graphic results depiction). Only participants with a stronger increase in self-reported anger in the aggression session compared to the control session were included as donors.

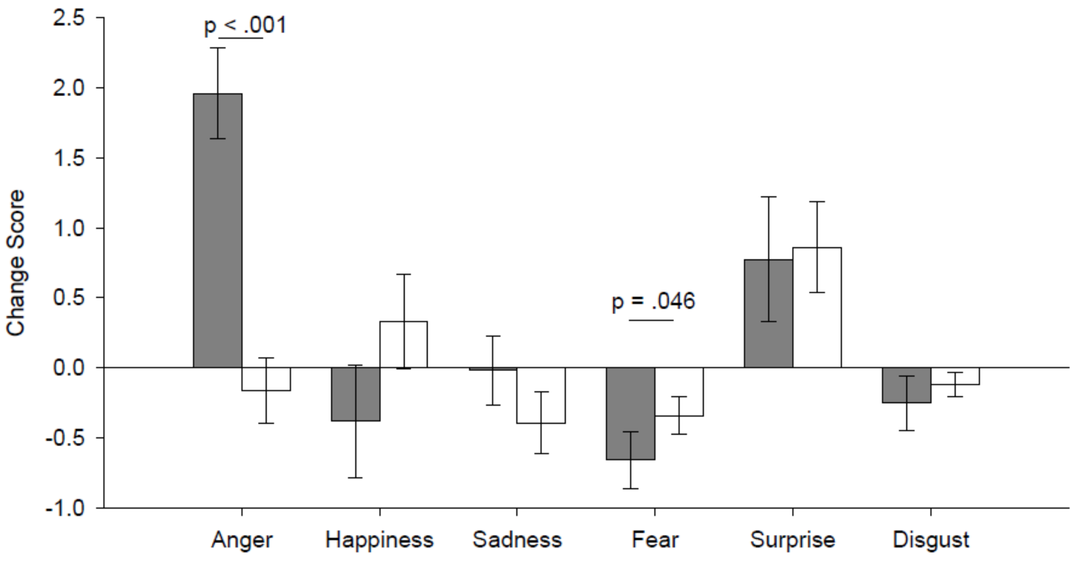

Figure S2. Donors’ mean (+ SEM) change of self-reported basic emotions (t5 – t1) within the aggression session (grey bars) compared to the control session (white bars).

Table S1. Change of the donors' emotional state during the sweat donation sessions.

Emotion	Change during aggression session		Change during control session	
	M	SD	M	SD
Anger	1.96	1.88	-0.16	1.34
Disgust	-0.25	1.13	-0.12	0.51
Fear	-0.66	1.17	-0.34	0.79
Happiness	-0.38	2.34	0.33	1.96
Sadness	-0.02	1.44	-0.39	1.29
Surprise	0.78	2.60	0.86	1.89

Notes: M = mean, SD = standard deviation. The "increase during session" is defined as the difference value between the emotional state after the session and before the session. Range: 0-10.

Table S2. Analyses of variance of the change of donors' emotional state during the sweat donation sessions (SESSION x DONOR's GENDER).

Effect	Anger	Disgust	Fear	Happiness	Sadness	Surprise
SESSION	AS > CS***		AS < CS*			
DG						
SESSION x DG	AS > CS in MP***<br>AS > CS in FP**					

Notes: SESSION: AS = aggression session, CS = control session, DG = Donors' gender: FP = female participants, MP = male participants, *** $p \leq .001$, ** $p \leq .01$, *$p \leq .05$.

In order to detect changes in testosterone secretion in response to aggression induction, saliva samples were obtained at five time points throughout the aggression session and the control session (figure S1, analysed by means of "Testosterone Saliva Kits", Tecan IBL international, Hamburg, Germany). Due to a modification of the Saliva Kits by the distribution company, only data of the aggression session could be analysed. Data of 4 donors were excluded from testosterone analysis due to being extreme outliers (with values exceeding the upper quartile by 3 times the interquartile range). The remaining 30 donors showed an increase (M = 9.28 pg/ml, SD = 24.26 pg/ml, mean change score of t2, t3, t4 relative to baseline) in salivary testosterone during being primed with aggressive video content and during the PSAP ($t(29) = 2.09$, $p = .045$, one-sample t-test against 0), while their

testosterone level returned to baseline when the PSAP had ended (M = -1.38 pg/ml, SD = 27.29 pg/ml, change score of t5 relative to baseline; see figure S3).

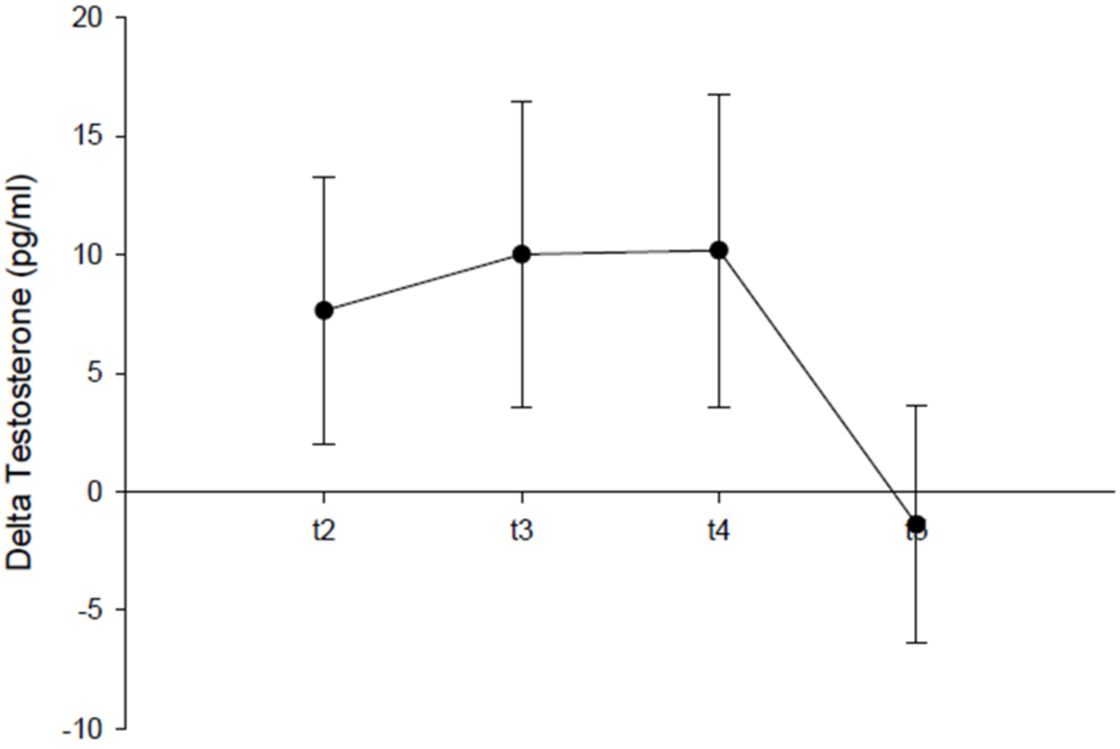

Figure S3. Donors’ mean (± SEM) changes of testosterone level in reference to baseline (t1) throughout the aggression session; t2 = following aggression priming, t3 = following the first block of the Point Subtraction Aggression Paradigm (PSAP), t4 = following the second block of the PSAP, t5 = following the third block of the PSAP/ end of the session.

The donors’ heart rate was sampled using a mobile pulse monitor (Omron R4 Plus Intellisense, Omron Medizintechnik Handelsgesellschaft mbH, Mannheim, Germany) on three occasions in each session (t1, t2, t5, see fig. S1). Donors’ mean (t2, t5) baseline-corrected heartrate decreased during the control session, but did not change during the aggression condition (Session: $F(1, 32) = 13.18$, $p = .001$, $\eta^2_p = .29$, Power = 0.94; effects including gender were not significant, all ps > .10; for mean values see table S3 in the supplementary material).

Table S3. Donors' mean (t2, t5) baseline-corrected heartrate during the sweat donation sessions.

Group	Aggression session		Control session	
	M [BPM]	SD [BPM]	M [BPM]	SD [BPM]
Male donors	1.32	5.61	-3.12	3.86
Female donors	-0.18	6.80	-6.00	6.37
All donors	0.57	6.18	-4.56	5.39

Notes: M = mean, SD = standard deviation, BPM = beats per minute.

PART B: MAIN EXPERIMENT

1. Methods

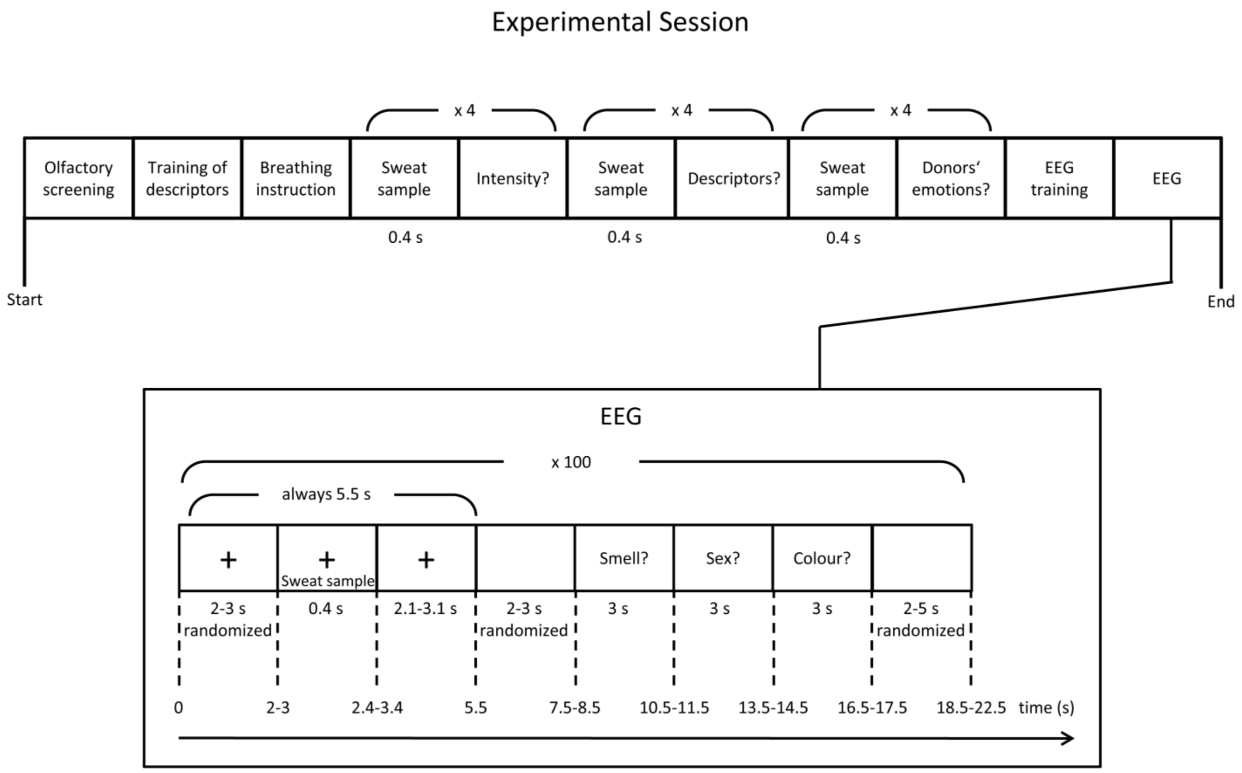

Figure S4: Time course of the experimental session. First, a brief olfactory screening was carried out. Then the large verbal descriptor list was trained and afterwards participants were instructed and trained on the velopharyngeal closure technique. Then, the odour ratings (intensity, verbal descriptors, donors' emotions) were obtained. Finally, the EEG and the detection rates were recorded.

2. Results

Detection rates and stimulus ratings

Table S4. Stimulus detection

Group	Male aggression sweat		Male control sweat		Female aggression sweat		Female control sweat	
	M [%]	SD	M [%]	SD	M [%]	SD	M [%]	SD
Male participants	57.74	30.34	55.13	26.50	49.39	27.91	51.13	29.22
Female participants	62.08	30.14	49.28	29.32	50.40	30.22	43.52	27.04
All participants	60.00	29.99	52.08	27.87	49.92	28.83	47.17	28.07

Notes: M = mean, SD = standard deviation (+/-).

Table S5. Assessments of donors' gender

Group	Male aggression sweat		Male control sweat		Female aggression sweat		Female control sweat	
	M [%]	SD	M [%]	SD	M [%]	SD	M [%]	SD
Male participants	50.44	19.46	44.57	16.65	52.82	17.51	55.20	16.52
Female participants	54.42	14.38	50.60	19.92	53.50	13.10	51.03	14.80
All participants	52.51	16.94	47.71	18.49	53.17	15.21	53.03	15.62

Notes: M = mean, SD = standard deviation (+/-).

Table S6. Odour intensity ratings

Group	Male aggression sweat		Male control sweat		Female aggression sweat		Female control sweat	
	M	SD	M	SD	M	SD	M	SD
Male participants	3.70	2.23	3.17	2.04	3.04	1.92	2.61	1.62
Female participants	3.60	2.29	2.88	1.90	2.40	1.94	2.76	1.92
All participants	3.65	2.24	3.02	1.95	2.71	1.94	2.69	1.76

Notes: M = mean, SD = standard deviation. Range: 1-9.

Table S7. Suspicion of donors' emotional state

Ascribed Emotion	Male aggression sweat		Male control sweat		Female aggression sweat		Female control sweat	
	M	SD	M	SD	M	SD	M	SD
Anger	1.84	1.91	1.77	2.09	1.78	2.02	1.78	2.12
Fear	2.57	2.64	1.56	1.55	1.89	2.16	1.58	2.09
Happiness	1.28	1.61	2.28	2.48	2.18	2.28	1.98	2.25

Notes: M = mean, SD = standard deviation. Range: 0-10.

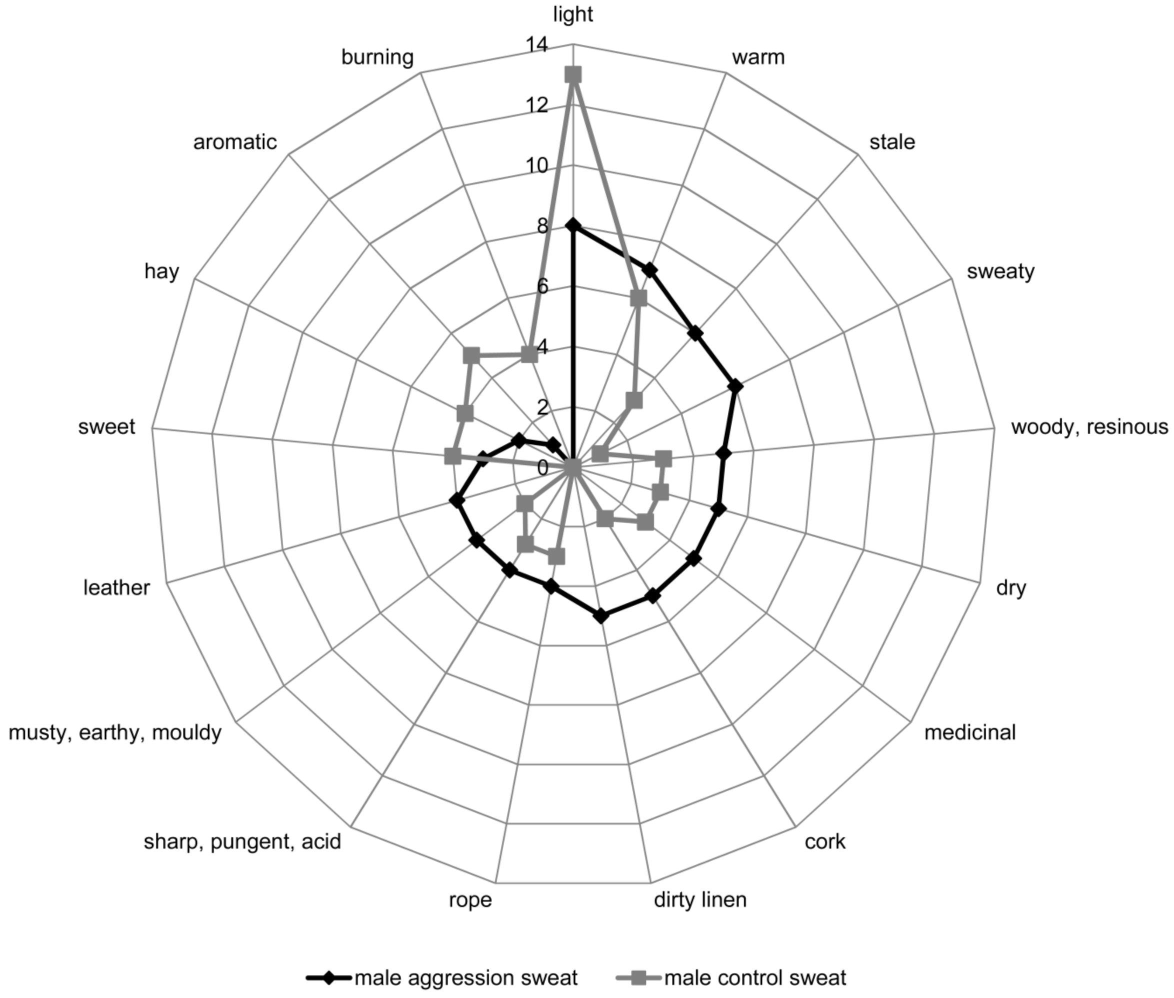

Figure S5. Frequency of verbal descriptors assigned to male sweat samples. Only descriptors chosen at least 4 times to describe one of the samples are depicted.

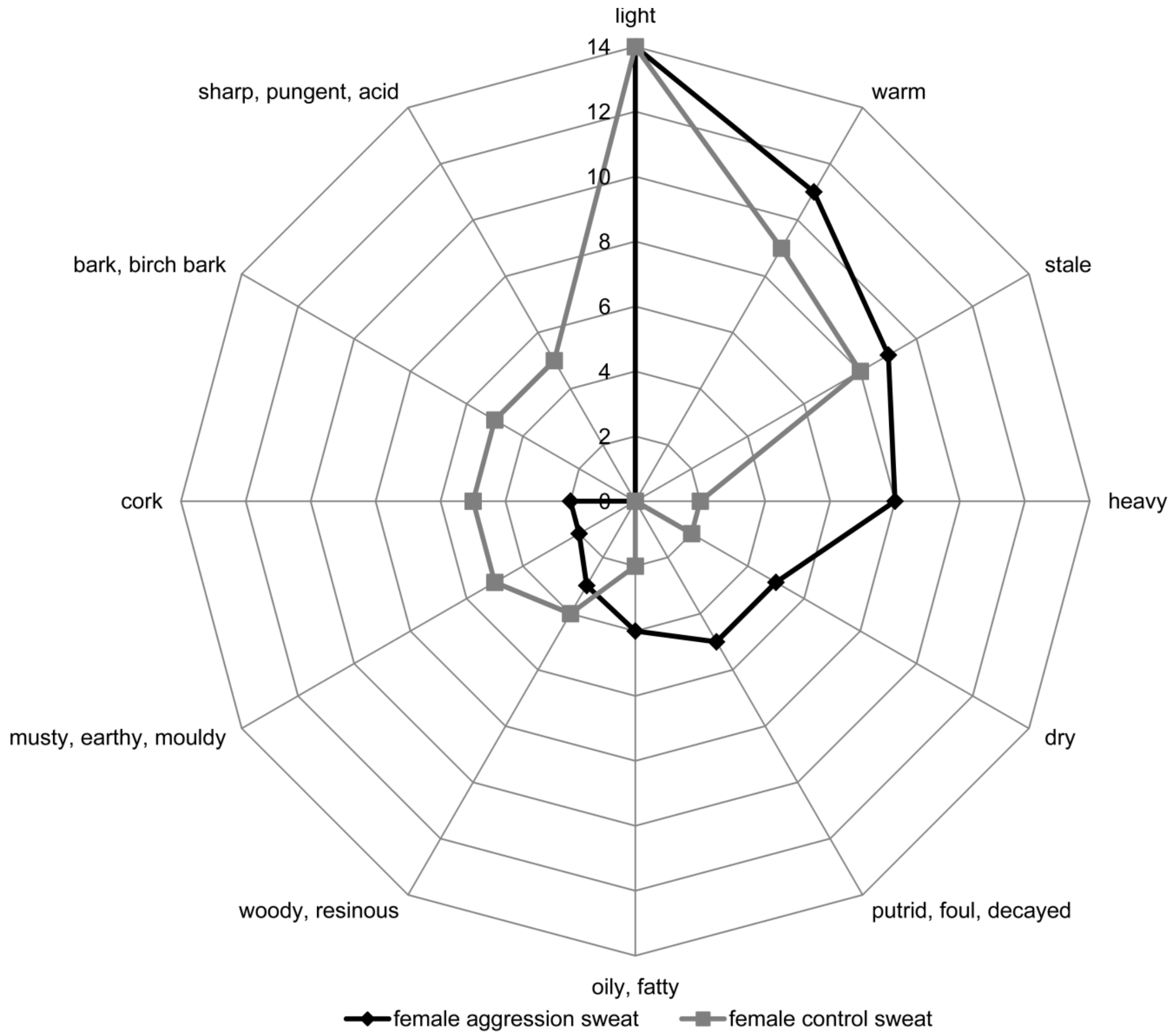

Figure S6. Frequency of verbal descriptors assigned to female sweat samples. Only descriptors chosen at least 4 times to describe one of the samples are depicted.

Psychophysiology

Local distribution of CSERP components

Amplitudes

P2

Independent of the sagittal level, the P2 amplitude appears largest at midline as compared to left and right electrode pools (TRANS: $F(2, 92) = 41.44$, $p < .001$, $\eta^2_p = .47$, Power = 1.00; SAG x TRANS; $F(4, 184) = 5.67$, $p = .002$, $\eta^2_p = .11$, Power = .98; nested effects: left vs. midline: $t(47) = 6.62$, $p < .001$; midline vs. right: $t(47) = 7.08$, $p < .001$; midline: M = 2.71 µV, SD = 2.16, left: M = 1.26 µV, SD = 1.36; right: M = 1.36 µV, SD = 1.41). The maximum P2 amplitude can be detected above central-midline (M = 3.17 µV, SD = 2.41) scalp areas (posterior-midline: M = 2.69 µV, SD = 2.39, anterior-midline: M = 2.27 µV, SD = 2.02; SAG x TRANS; nested effects: central-midline vs. anterior-midline: $t(47) = 4.30$, $p < .001$; central-midline vs. posterior-midline: $t(47) = 5.27$, $p < .001$; due to this interaction, the main effect SAG is invalidated).

P3-1

Similar to the P2 component, the P3-1 amplitude appears with a dominance across midline scalp regions at all steps of the sagittal level (TRANS: $F(2, 92) = 46.48$, $p < .001$, $\eta^2_p = .50$, Power = 1.00; SAG x TRANS; $F(4, 184) = 7.92$, $p < .001$, $\eta^2_p = .15$, Power = 1.00; nested effects: left vs. midline: $t(47) = 6.95$, $p < .001$; midline vs. right: $t(47) = 7.17$, $p < .001$; left: M = 1.88; SD = 1.67; midline: M = 3.81 µV, SD = 2.71 µV; right: M = 2.09 µV, SD = 1.82 µV). Above midline scalp areas, the P3-1 amplitude is largest within the central sagittal line (M = 4.50 µV, SD = 3.08 µ) compared to the anterior (M = 2.78 µV, SD = 2.46 µ) and posterior line (M = 4.15 µV, SD = 3.08; SAG x TRANS; nested effects: anterior-midline vs. central-midline: $t(47) = 6.32$, $p < .001$; anterior-midline vs. posterior-midline: $t(47) = 4.36$, p

< .001; central-midline vs. posterior-midline: t(47) = 3.37, p = .001). However, in contrast to the P2 amplitude, the P3-1 amplitude displays a parietal dominance above left and right sided electrodes (SAG x TRANS; nested effects: poster-left vs. central-left: t(47) = 2.72, p = .009; posterior-right vs. central-right: t(47) = 2.50, p = .016; due to the interaction SAG x TRANS, the main effect SAG is invalidated).

P3-2

The local distribution of the P3-2 component mainly resembles the distribution of the P3-1 component. It is largest above midline as compared to left and right electrodes (TRANS: F(2, 92) = 36.62, p < .001, η^2_p = .44, Power = 1.00; SAG x TRANS; F(4, 184) = 5.79, p = .002, η^2_p = .11, Power = .98; nested effects: left vs. midline: t(47) = 6.26, p < .001; midline vs. right: t(47) = 6.43, p < .001; midline: M = 3.61 µV, SD = 2.72; left: M = 1.78 µV, SD = 1.71; right: M = 1.92 µV, SD = 1.85).

Similar to the P3-1 component, the P3-2 amplitude is larger across central midline (M = 4.26 µV, SD = 3.17 µV) electrode pools compared to anterior-midline (M = 2.57 µV, SD = 2.46 µV) and posterior-midline electrode pools (M = 4.01 µV, SD = 3.09 µV; SAG x TRANS; F(4, 184) = 5.79, p = .002, η^2_p = .11, Power = .98; nested effects: anterior-midline vs. central-midline: t(47) = 5.67, p < .001; anterior-midline vs. posterior-midline: t(47) = 4.21, p < .001; central-midline vs. posterior-midline: t(47) = 2.24, p = .030). However, at left and right sided electrodes the P3-2 component shows a parietal dominance (SAG x TRANS; nested effects: poster-left vs. central-left: t(47) = 2.50, p = .016; posterior-right vs. central-right: t(47) = 2.29, p = .027; due to the interaction SAG x TRANS, the main effect SAG is invalidated).

Latencies

The latencies of either component are unaffected by transversal and sagittal position factors (all ps > .055).

Overview on all ANOVA CSERP results

Table S8. ANOVA results of CSERP amplitudes: Significant main effects, interactions, and single comparisons.

Effect	P2	P3-1	P3-2
EMO			AS > CS*
DG		MS > FS*	MS > FS**
SAG	c > a***<br>p > a**	c, p > a**	c, p > a***
TRANS	m > l, r***	m > l, r***	m > l, r***
DG x TRANS			MS > FS in l**, m***, r*
PG x TRANS		FP > MP in m*	FP > MP in m*
SAG x TRANS	c, p > a in l***<br>c > a, p in m***<br>p > a in r**<br>c > a in r***<br><br>m > l, r in a, c, p***	c, p > a in l, m, r***<br>p > c in l**, r*<br>c > p in m***<br><br><br>m > l, r in a, c, p***	c, p > a in l, m, r***<br>p > c in l, r*<br>c > p in m*<br><br><br>m > l, r in a, c, p***
EMO x DG	 <br>MS > FS in AS*	AS > CS in MS**<br><br>MS > FS in AS**	AS > CS in MS**<br><br>MS > FS in AS***
EMO x DG x PG		AS > CS in MS in FP**<br><br><br>MS > FS in AS in FP***<br><br>FP > MP in CS in FS*	AS > CS in MS in FP**<br>AS > CS in FS in MP*<br><br>MS > FS in AS in FP***<br><br>FP > MP in CS in FS*
EMO x DG x TRANS		AS > CS in MS in l**, m*<br><br>MS > FS in AS in l***, m***, r*	

Notes: EMO = Emotion: AS = aggression sweat, CS = control sweat, PG = Participants' gender: FP = female participants, MP = male participants, DG = Donors' gender: MS = male sweat, FS = female sweat, SAG = Sagittal line: a = anterior, c = central, p = posterior, TRANS = Transversal line: l = left, m = midline, r = right, ***$p \leq .001$, **$p \leq .01$, *$p \leq .05$.

Table S9. ANOVA results of CSERP latencies: Significant main effects, interactions, and single comparisons.

Effect	P3-1 latency	P3-2 latency
EMO x DG		AS > CS in MS**<br>CS > AS in FS*<br><br>MS > FS in AS*<br>FS > MS in CS*
EMO x DG x TRANS	AS > CS in MS in l**	

Notes: EMO = Emotion: AS = aggression sweat, CS = control sweat, PG = Participants' gender: FP = female participants, MP = male participants, DG = Donors' gender: MS = male sweat, FS = female sweat, TRANS = Transversal line: l = left, m = midline, r = right, *** $p \leq .001$, ** $p \leq .01$, *$p \leq .05$.

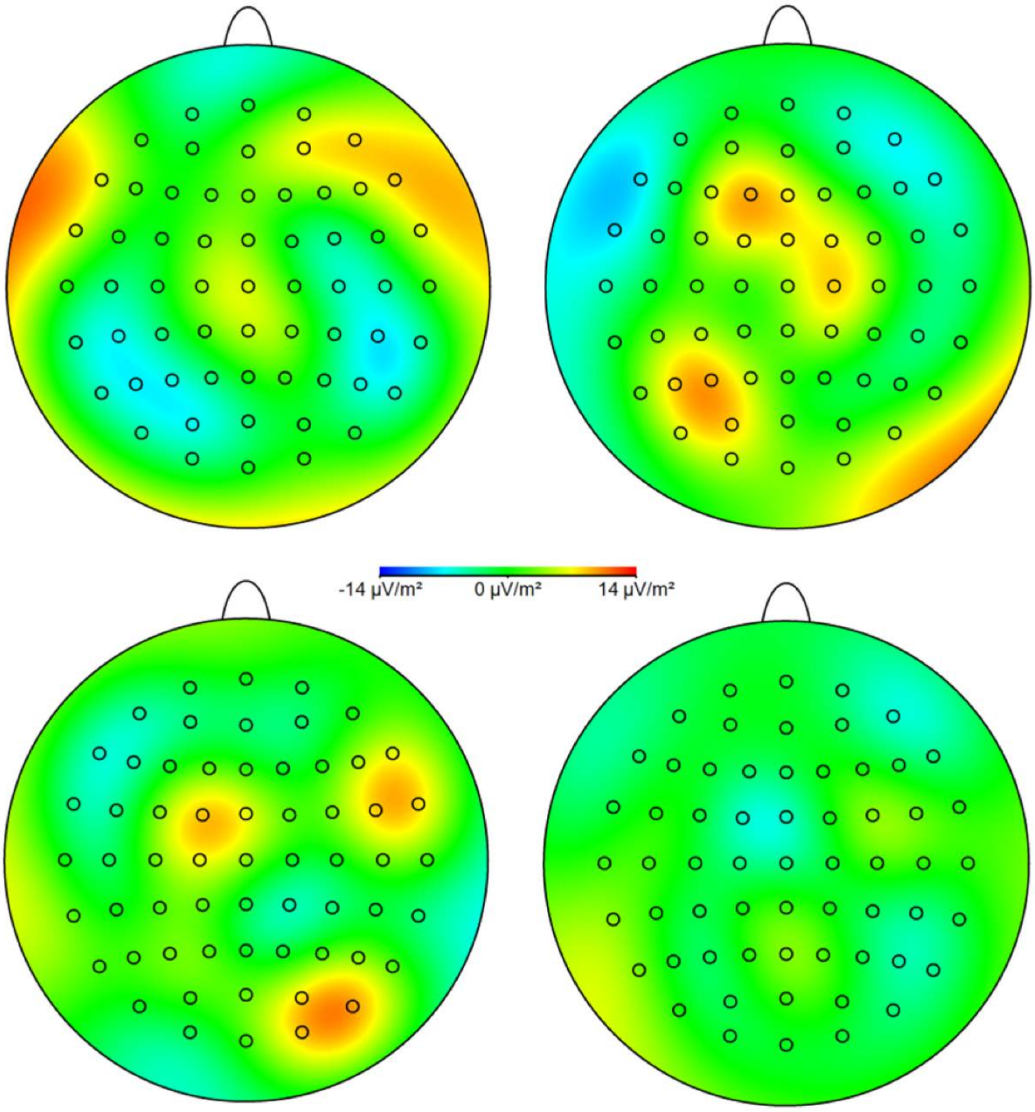

Figure S7. Current source density (CSD) difference maps (two dimensional smoothing for a view across all electrodes) of male (left column) and female participants (right column) based on differential CSERPs of responses to male aggression minus male control sweat (upper row) and female aggression minus female control sweat (lower row) at the time of the total mean P3-1 latency peak (810 ms). Red colours represent cortical activation (neuronal sources) and blue colours represent cortical deactivation (neuronal sinks).

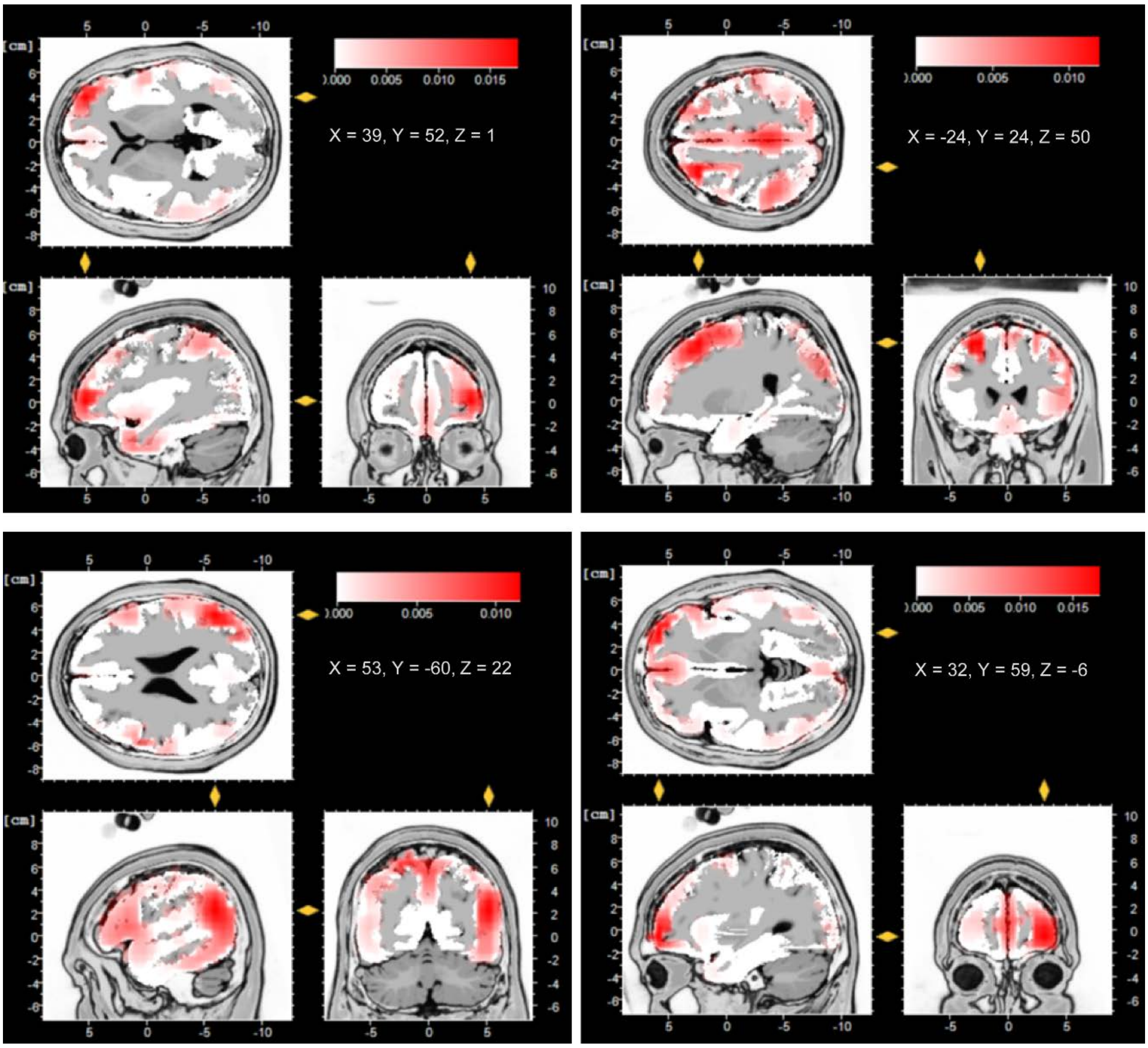

Figure S8. Low Resolution Electromagnetic Tomography (LORETA) maps depicting the location of the maximum current density (in μA/mm^2) at the time of the total P3-1 latency (810 ms) of men responding to male aggression sweat (in contrast to male control sweat, upper left), women responding to male aggression sweat (in contrast to male control sweat, upper right), men responding to female aggression sweat (in contrast to female control sweat, lower left), and women responding to female aggression sweat (in contrast to female control sweat, lower right).

References

[1] Cherek, D. R. 1981 Effects of smoking different doses of nicotine on human aggressive behavior. *Psychopharmacology (Berl.)* **75**, 339-345. (doi:10.1007/bf00435849).

[2] Carré, J. M. & McCormick, C. M. 2008 Aggressive behavior and change in salivary testosterone concentrations predict willingness to engage in a competitive task. *Horm. Behav*. **54**, 403-409. (doi:10.1016/j.yhbeh.2008.04.008).
